# Computational Models for Transplant Biomarker Discovery

**DOI:** 10.3389/fimmu.2015.00458

**Published:** 2015-09-08

**Authors:** Anyou Wang, Minnie M. Sarwal

**Affiliations:** ^1^Department of Surgery, Division of MultiOrgan Transplantation, University of California San Francisco, San Francisco, CA, USA

**Keywords:** transplant, model, theory, computation, bioinformatics, rejection

## Abstract

Translational medicine offers a rich promise for improved diagnostics and drug discovery for biomedical research in the field of transplantation, where continued unmet diagnostic and therapeutic needs persist. Current advent of genomics and proteomics profiling called “omics” provides new resources to develop novel biomarkers for clinical routine. Establishing such a marker system heavily depends on appropriate applications of computational algorithms and software, which are basically based on mathematical theories and models. Understanding these theories would help to apply appropriate algorithms to ensure biomarker systems successful. Here, we review the key advances in theories and mathematical models relevant to transplant biomarker developments. Advantages and limitations inherent inside these models are discussed. The principles of key ­computational approaches for selecting efficiently the best subset of biomarkers from high-­dimensional omics data are highlighted. Prediction models are also introduced, and the integration of multi-microarray data is also discussed. Appreciating these key advances would help to accelerate the development of clinically reliable biomarker systems.

## Introduction

In the new era of biomedical research, it is being increasingly recognized by funding agencies and journals that traditional hypothesis-driven research alone cannot provide the rapid and incremental advances needed to change the current clinical practice management for transplant patients, so as to positively impact long-term graft outcomes. In addition, given that organ transplantation is an orphan disease, there are few if any focused efforts for discovery of new immunosuppressive drugs for transplant recipients. In fact, the number of Food and Drug Administration (FDA) approved drugs has been relatively constant to about 20 drugs per year, yet the cost of drug discovery has ramped up ($138 million in 1975 to $1.3 billion in 2006), and the rate of new drug production by a pharmaceutical company generally follows a Poisson distribution and is constant (about 2–3 drugs per year at most) (1). This constant rate of output is often blamed on the traditional hypothesis-driven research model, primarily because hypotheses derived from complex experimental models often do not translate to human pathology. Hence, there is a growing need to harness “big data” at the RNA/protein/metabolite/antibody/DNA level to get novel insights into interlinked global processes that have been hitherto poorly understood. With this direction, comes the companion need to develop and apply the right computational tools to harness this data and interlink it to the entire electronic medical record (EMR) in an identified, regulated process.

**Figure 1:**
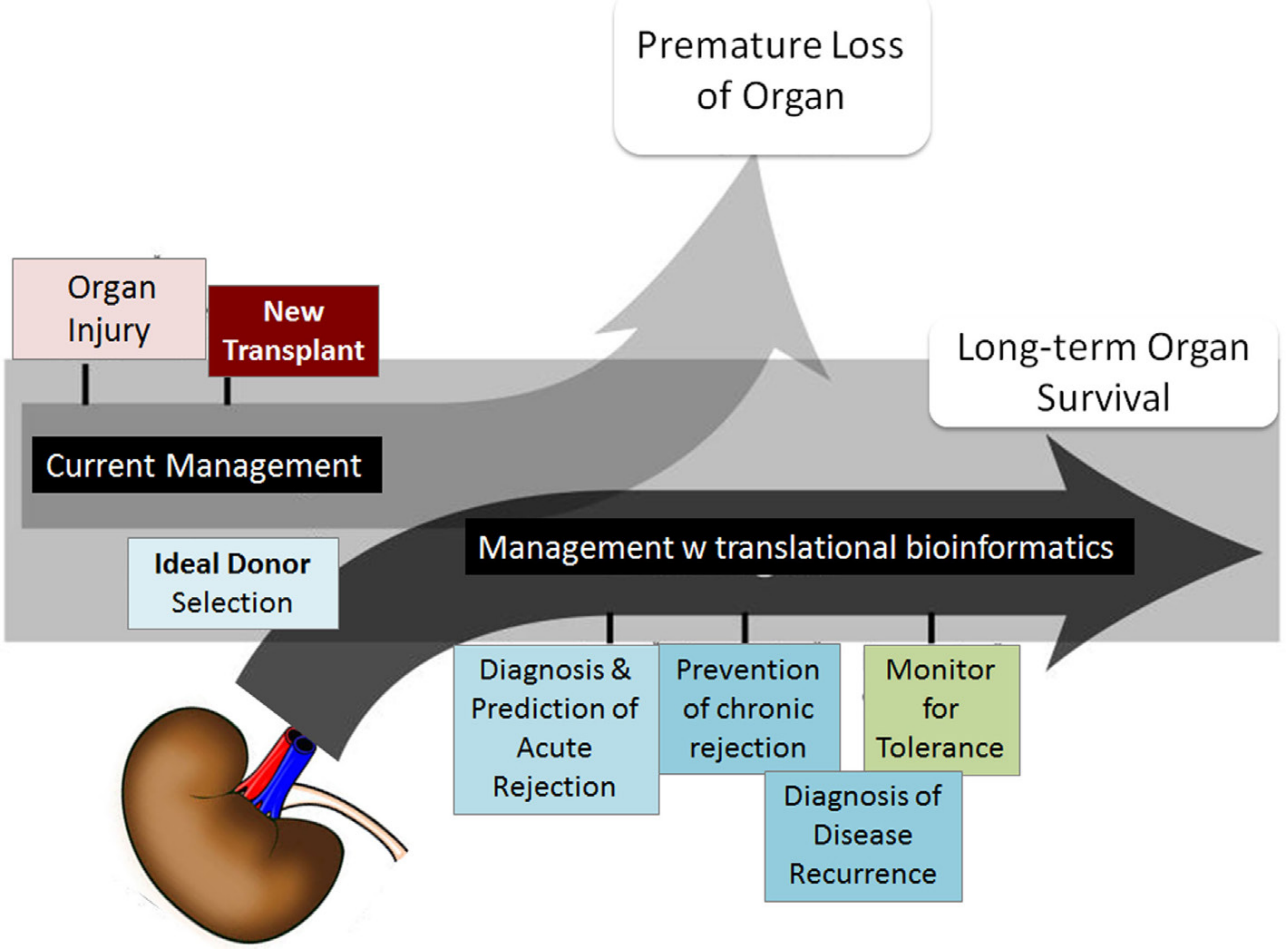
**Transplant fields require computations**. The boxes show the areas of investigation needed by translational computational methods to advance organ transplant management. Figure adapted from Ref. (6).

Although short-term survival rates of grafts have increased, long-term graft survival rates have shown little improvement (2, 3). Five-year graft survival for transplanted organs varies from 43% for lung to 78% for kidney, highlighting the need for improved analysis of post-transplant injury pathways. There is a desperate urgency to advance the field of organ transplantation through improved monitoring by (a) the discovery of informative biomarkers, specific and sensitive to phenotypes of injury and acceptance, and (b) through improved algorithms and/or drugs for treatment with targeted efficacy and reduced toxicity (4, 5). Many single gene/protein pathway studies have shown associative and mechanistic insights into animal and restricted human sample studies, but the field has stalled with regard to the additional exponential insights needed at a genome-wide level to develop significant improvements in biomarker discovery for diagnosis/prediction and to evaluate the role of novel pathways for improved rational drug design as it applies to organ transplantation. In this review, we focus on the application of different computational approaches to mine high-dimensional human data in transplantation with a view to changing current clinical practice and patient management. Some of the critical requirements that the transplantation process needs to fulfill with this meta-data approach are highlighted in Figure 1.

**Figure 2:**
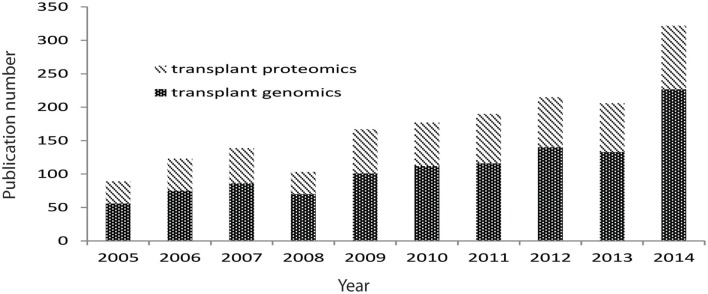
**Pubmed publications on transplant genomics and proteomics paper over last 10 years**. Data were extracted from Pubmed by searching transplant and genomics or transplant and proteomics.

Biological experimental tools that explore genome-wide profiling referred as “omics” provide promising pathways to investigate transplant biology, and they have been increasingly applied in transplantation, with the number of generated data tripling over last decade (Figure 2). These omics technologies (e.g., functional genomics for RNA analysis, proteomics for protein and peptide analysis, metabolomics for metabolite analysis, and antibiomics for HLA- and non-HLA-antibody analysis) also provide “big data” that contains high-dimensional variables. Harnessing the “big data” to low dimensional variables could generate small sets of biomarkers for diagnostic tools, which detect and predict transplant injury as well as discriminate different causes of injuries. However, these omics data are generally complex, due to its inherent high-dimensional complexity, platform differences, hybridization variations, and different data scales. These complexities challenge scientists to directly extract biologically valid and clinically useful information by selecting, generating, and using the appropriate computational tools to meet the demands of the composition of the input data.

Decomposing the complex omics datasets to derive biomarkers often requires customization of computer algorithms and software. Limitations and pitfalls inherent inside these software tools, such as biased *p*-value estimation, over-estimated prediction accuracy, could derail the successful selection of biomarkers due to enhanced false positives and negatives. Thus, applying appropriate algorithms to decompose the complexity requires an understanding of the theories and principles behind these algorithms. Here, we review key advances of theory and computational models relevant to transplant biomarker development. Understanding these key advances would help to master the wave of biomarker development and to develop novel reliable biomarker systems (4, 7–9).

## High-Dimensional Data Applications in Transplantation

Gene expression microarrays have been the most commonly used high-throughput technology in transplantation (10, 11). Microarrays were applied to biomedical science in 1995, and the first landmark study of kidney biopsy microarrays was published in 2003 [Sarwal (12), *NEJM*], uncovering for the first time molecular heterogeneity in acute rejection that was far greater than previously understood by histology alone and a pivotal role for B cells in steroid-resistant late post-transplant rejections occurring secondary to treatment non-adherence. There are an increasing number of human studies in the public domain profiling biopsy, bronchoalveolar lavage, and blood and urine samples from different organ transplant recipients, with phenotypes defined by matching graft biopsies as acute rejection, chronic injury, recurrent glomerulonephritis, viral nephritis, operational and induced tolerance, and drug toxicity. Transcriptional profiling of peripheral blood as a correlate of intragraft events has been successfully applied in the IMAGE Study in heart transplantation (13) and in the SNSO1 (14) and AART (15) studies in kidney transplantation and for the detection of chronic graft vs. host disease in bone marrow transplantation (16). Pathogenesis-based transcripts (PBT) expression panels have been inferred from mouse experiments and applied to human transplant expression patterns in an effort to develop correlates of histopathological lesions in renal transplant biopsies (17).

A major challenge in transplantation is the life-long administration of immunosuppressive drugs with multiple side effects. Calcineurin inhibitors are associated with nephrotoxicity, which in turn can contribute to long-term graft failure, along with opportunistic infections. To better understand the mediators of calcineurin inhibitor toxicity, selected patients from the BENEFIT trial (Vincenti, NEJM), had their 1-year protocol biopsies profiled against gene-sets selected after loading cyclosporine and tacrolimus on renal proximal tubular cells, as the *in vitro* model of calcineurin inhibitor toxicity. Patients receiving Belatacept and no calcineurin inhibitor agents demonstrated more immune reactivity, but reduced expression of profibrotic genes and increased expression of solute transporter genes, correlating with the preserved renal architecture seen in these patients (18). To better understand how to optimally dose patients with immunosuppressive drugs, operationally tolerant patients were profiled (19), and informative genes were used to identify patients on full dose immunosuppression that may benefit from safe immunosuppression wean (19), after controlling for multiple clinical variables and confounders.

High-throughput technologies have also been expanded to study the role of microRNAs (miRNAs) in graft rejection in peripheral blood (20) and the allograft (18), and suggests that intragraft changes in miRNA levels are explained by the burden and composition of infiltrating cells in the course of injury.

The introduction of high-density protein arrays as allowed for the evaluation of serological responses to ~9,000 human full-length proteins on a single slide. This technology was used to understand the differential immunogenicity of different tissue compartments of the transplanted kidney (21), and identified that the renal outer cortex, glomerulus, and the deep pelvis antigens mount new autoantibody responses after organ engraftment, most of which may not be pathogenic. In addition, this technology was also used to evaluate the identity of novel non-HLA antibodies in patients with HLA-antibody-negative acute renal transplant rejection, and identified a novel target, Kinase C-ζ (PKCζ), as a dysregulated epitope in severe allograft injury (22). Additionally, using protein microarrays, Angiotensinogen and PRKRIP1 were identified as biomarkers of chronic kidney injury, with correlative results with hypertension in patients with high-antibody titers. These results suggested for the first time that autoantibodies are raised against previously unknown antigenic targets in the transplanted organ, which are likely exposed to the immune system of the recipient in the process of cellular damage in the organ (23).

Recent advances in small molecule identification technologies (e.g., mass spectrometry, surface enhanced laser desorption/ionization, liquid chromatography/mass spectrometry, nuclear magnetic resonance) have given rise to the application of proteomics, peptidomics, and metabolomics to transplantation. Urine is a rich biofluid source for biomarker discovery in organ transplantation. Shotgun proteomics provides us with a map the entire urinary proteome (24) in health and transplant injury states. Smaller fragments of the urinary peptidome, consisting of degraded byproducts of intact proteins by enzymatic cleavage, also provide insights into the perturbations in chemical balance during kidney injury (25, 26). Metabolomics has been used for identifying graft injury as well as for monitoring drug toxicity (27–29).

## Computational Challenges and Approaches for Selecting Biomarkers from High-Dimensional Data

The integration of hypothesis generation by high-dimensional data analysis is poised to fulfill the current unmet needs in organ transplantation, which relates to poor long-term survival despite improvement in short-term outcomes, the need for life-long immunosuppressive medications and their associated morbidities, and the lack of non-invasive markers for monitoring and predicting graft injury, superior to current standards of monitoring. Computational biology expands from the traditional molecular biological method of studying pair-wise interactions into a network-based approach by integrating individual components to model a complex system, thus beginning to understand disease at the level of regulatory pathways in tissues and organs, even in whole organisms, while also accounting for dynamics within regulatory networks. A large number of computational approaches have been developed to generate co-expression networks from protein binding data (30), functional annotations (31), and drug activity (32). Using these approaches, it has been shown that such networks have properties that are not otherwise discernable from the relations themselves, and have preferential connectivity that results in “hub” nodes, which are molecules that connect to a larger number of other molecules (33).

To date, a large number of biomarkers have been identified for various post-transplant conditions as markers of an ongoing injury (effect markers) or related to the actual causes of the injury (causal markers). Development of new drugs that reduce drug toxicity and chronic rejection requires identification of causal markers that can be targeted for novel therapeutics. The use of systems and computational biology is the critical next step for deeper understanding and the identification of causal markers of graft injury in transplantation.

One of the reasons for the limited impact of the high-throughput studies in transplantation relates to low number of individuals and samples used resulting in lack of sufficient independent validation. As described in our previous review (6), searching the NCBI GEO for microarray studies in humans described with the term “transplant” yields 69 experiments, of which only 16 have more than 50 samples and only 6 have more than 100 samples. These numbers are even more disappointing when put into the context that these experiments are divided among four different organs (lung, kidney, liver, heart) studying at least three different conditions (acute rejection, chronic rejection, tolerance). The sample limitations relate to sample availability and assay cost, both of which truncate greater enrollment. We addressed the sample availability shortcoming by performing meta-analysis by integrating smaller independent experiments, and customized algorithms were generated to deal with experiment-specific technical biases, such as microarray platform or hybridization protocol (6, 34). This approach allowed for the identification of a core of 12 genes (BASP1, CD6, CD7, CXCL9, CXCL10, INPPD5, LCK, NKG7, PSMB9, RUNX3, TAP1, ISG20), called the common immune response module, which was a similarly dysregulated set of genes in acute rejection across tissue source; these genes were all upregulated in kidney, heart, liver, and lung rejection across 236 microarrays downloaded from GEO. Their biological relevance in graft rejection was further tested by repositioning two drugs against LCK (Dasatinib) and CXCL10 (Atorvastatin) in a murine heart transplant model of rejection; thus suggesting that FDA-approved drugs for indications other than transplant immunosuppression may be repositioned across the remainder of the gene-set to identify new drug targets for organ transplant recipients. In another example of an integrative analysis, Chen et al. performed a meta-analysis using three transplant RNA microarray data sets from biopsies with kidney and heart acute rejection (35), and then the corresponding significant proteins (inferring gene/protein 1:1 mapping) coded for by these RNA were then screened as potential blood markers for acute rejection. This approach confirmed that three proteins (PECAM1, CD44, and CXCL9) were significantly over-expressed in blood samples in both kidney and heart transplant patients. These integrative approaches demonstrate that integration of data sets can reduce biological bias across experiments, experimenters, platforms, and tissue source, while allowing for the generation of novel hypotheses and drug repositioning.

Data integration can also be performed across different types of molecular measurements. Li et al. integrated antibody-level measurements from a protein array with renal compartment-specific gene expression data (21) and demonstrated that post-transplant serological responses observed using protein microarrays were specific to the transplanted organ and to specific organ compartments.

The efficient selection of biomarkers to limit *false negatives and false positives* is another challenge for high-dimensional data analysis. A typical microarray experiment produces approximately 50,000 data points per sample. An experiment with 50 samples will produce more than 2.5 million data points. Millions of data points are generated by SNP genotyping platforms for thousands of samples in a typical genome-wide association study. The amount of data generated increases again exponentially for next-generation sequencing population studies. Incorporation of computational skills into the curricula of transplantation training program needs to be a high priority, to arm the next-generation clinician scientist.

Various methods are used to limit false-positive/negative signals and reduce variables. These mainly use stepwise regression models (36, 37), principal component analysis (PCA) (38, 39), *T*-statistic, and correlation to clinical variables (7, 8, 40–43).

### Stepwise-based models

#### Traditional Stepwise Methods

Many regression models are available to select a particular set of independent variables and the commonly used methods are stepwise techniques (36, 37). Traditional stepwise selection alternates between forward and backward regression selection, in which variables are added or removed that meet a selection criteria setting for entry or removal, until a final subset of variables make the model saturated. However, this stepwise method has essential problems. It applies methods intended for one single hypothesis test to many tests, leading to results biased to a certain degree, such as higher in *R*^2^ (explained variation/total variation), lower in standard errors and *p*-values than the actual values, and as models they can be complex to develop.

Vorlat et al. used stepwise multiple regression and identified B-type natriuretic peptide (BNP) and age as the most important factors in evaluating outcomes after heart transplantation, after evaluating many variables, such as body mass index, age, BNP, norepinephrine dose, gender, and total ischemic time (37).

#### Lasso (Least Absolute Shrinkage and Selection Operator)

Lasso (least absolute shrinkage and selection operator) (44) is a penalized regression method for shrinkage and variable selection, and uses the equation:

$${\sum_{i=1}^{n}\left(y_{i}-\sum_{j}x_{ij}{\text{β}}_{j}\right)}^{2}+\lambda \sum_{j=1}^{p}\left|{\text{β}}_{j}\right|$$

*i* = 1, 2, …, *n* (*n* equivalent to sample size);
*j* = 1, 2, …, *p* (*p* equivalent to omics gene number);
*y_i_* = response variable of sample *i*, β*_j_* = coefficient for gene *j*, *j* = 1, 2, …, *p*, and *x_ij_* = observation value of sample *i* and gene *j*.

Lasso estimation actually introduces a penalized constraint to minimize the usual sum of squared errors to get solution. This penalization is estimated by $\sum |{\text{β}}_{\text{j}}|\leq s,$ sum of the absolute coefficients.

If *s* is set to a large number, it does not affect Lasso estimation that actually acts as a usual multiple linear least squares estimates. Then, a large number of genes might be selected as biomarkers. However, if *s* is small (*s* ≥ 0), Lasso works as shrunken least squares regression and then only a few genes would be selected as biomarkers. Lasso has several limitations. For example, the gene number (*p*) is usually large and sample size (*n*) is small. In this case, at most *n* genes are selected by Lasso before the model saturates. In addition, Lasso tends to select the biomarker with greater variance (44) and it might likely ignore some important genes in a correlated group.

#### Elastic-Net

To overcome the limitations existing in Lasso, elastic-net adds an additional quadratic part $\sum_{j}{\text{β}}_{j}^{2}\leq t$ to the penalization to make it work for both variable selection and shrinkage (45). Many modifications have been made to improve its prediction performance. Elastic-net and lasso are arguably the best methods so far for shrinkage and biomarker selection. Lasso and elastic-net were to select a best subset of 17 genes as biomarkers to predict the most informative acute rejection blood-based biomarkers (15).

### Modified *t*-statistic methods

#### Prediction Analysis of Microarrays

Prediction analysis of microarrays (PAM) has been commonly applied in transplant (40, 46, 47). PAM uses the following equation to determine if a gene is significant for classification:

$$d_{ik}=\frac{{\bar{x}}_{ik}-{\bar{x}}_{i}}{w_{k}\left(s_{i}+s_{0}\right)}$$

where $w_{k}={\left(1/n_{k}-1/n\right)}^{1/2}$

It actually looks like *t*-statistic formula, where *w_k_* × *s_i_* is the standard error of the numerator. The only modification is to add *s*_0_ as a fudge factor to avoid very large statistics for very small standard errors. Thus, PAM is a modified *t*-statistic to measure the difference between the mean of gene *i* in class *k* with the overall mean of gene *i*. A gene with a statistic of large absolute value discriminates one class from the rest. PAM then selects significant genes by then shrinking the *d_ik_* toward zero.

This measurement in PAM actually shrinks each gene toward its overall mean cross classes. After this shrinkage, all class centroids become more similar to each other than before. This might not help to improve the overall discriminant accuracy in omics data.

Reeve et al. used PAM to select the most significant genes from 186 microarrays to build a classifier system to predict acute rejection. These genes are mostly associated with interferon-gamma-inducible or cytotoxic T-cell associated, such as CXCL9, CXCL11, GBP1, and INDO (47).

#### ClaNC

To circumvent the PAM limitations, an alternative classifier called ClaNC (42) has been developed. ClaNC uses standard statistics to select genes and does not shrink centroids, and it also selects class-specific gene and allows a gene to be active only in one class. Kurian et al. used this algorithm to select 200 biomarkers from genome-wide gene expression profiling and created a discriminant system for classifying three phenotypes in kidney transplantation (48).

### Principal component analysis

Principal component analysis method selects biomarkers based on an eigengene score (38), $\text{score=|cor}\left(x_{i},E\right)|,\left|\text{cor}\left(x_{i},E\right)\right|$ is the absolute value of Pearson correlation coefficient, where *x_i_* is a vector of gene *i*, and *E* is a eigenvalue.

### Genes selected by correlating with clinical variables

A subset of genes can also be selected by correlating gene expression level and clinical variables, such as patient survival or graft loss. This correlation can be measured by univariate Cox proportional hazards scores that derived from Cox regression model. Genes that pass the interquartile range (IQR) filter are considered as significant (40, 41).

## Evaluation of Models and Biomarkers Requires Robust Validation

Many biomarker systems published to date for transplant fail in the real world, partially due to lack of robust model validation. Models usually should be subject to cross-validation, a technique for evaluating the performance of predictive models, like linear models for discriminating acute rejection against stable samples, with the use of independent samples in the different subsets. Different types of cross-validations are available. *Repeated random sub-sampling validation* randomly splits all samples into subsets of samples, a training set, and validating set. The training subset is used to fit a model and the validating set is for examining predictive accuracy. A large iteration of splits (e.g., 10,000) is usually run to avoid splitting sample bias. The accuracy is calculated by the average of all iterations. *k-fold cross-validation* randomly splits total samples into *k* subgroups with equal size. One out of *k* subgroups is treated as testing validation and the remaining *k* − 1 subgroups are used as training data to train models. The whole process is run *k* times (*k* folds). Each time, each of the *k* subgroups is used as validation but each group is used only once. The sensitivity and specificity can be calculated by combined result from each run. We have extensively used these systems of study (15, 19), and the process of robust cross-validation has also been extensively reviewed in a recent publication by Roedder et al. (49).

The binary classified performance of an entire biomarker system should typically be evaluated by *receiver operating characteristic curve*, ROC curve. ROC is a graphical plot of the true positive rate (sensitivity) against specificity or false positive rate (1-specificity) at various threshold settings. The sensitivity is equal to the proportion of correctly classified positive observations, and the specificity is calculated as the proportion of correctly classified negative observations. Area under the curve (AUC) serves as estimated index of overall accuracy and serves a useful practice to compare different ROCs, and it is usually plotted within ROC curve. The biomarker panels developed for graft rejection and tolerance in recent studies provide ROC curves of >85% (15, 19).

## Prediction Models for Biomarker Risk Analysis

One of the most interesting goals in developing biomarker systems is to predict and monitor the phenotype outcome of transplantation. For example, selecting biomarkers from short-term data (e.g., 3 months biopsy profiling) may be associated with a phenotype of long-term (e.g., 1-year graft chronic injury) outcomes. Predictive models have been applied to reach this purpose. Although all machine learning models have predictive functions, such as Support Vector Machine (38, 39) and LASSO described above, two out of them, linear discriminant analysis (LDA) and logistic regression, have been widely applied in transplant biomarker systems to classify the discrete classes of variables (40, 48, 50, 51).

### Linear discriminant analysis

Linear discriminant analysis has been widely applied to transplant biomarker development (40, 48). LDA is a classifier (52) that classifies samples to their nearest given centroid. Assuming that we have *k* classes with prior probabilities π*k*, then LDA can be defined below:

$$\hat{y}=\text{arg}\;{\text{min}}_{k}\left\{{\left(x-{\text{μ}}_{k}\right)}^{T}{\text{Σ}}^{-1}\left(x-{\text{μ}}_{k}\right)-2\text{log}\left(\pi_{k}\right)\right\}$$

where $∥x-u{∥}^{2}$ is the square of the Mahalanobis distance between sample value (*x*) and centroid or mean (*u*).

### Logistic regression

Logit link function can be understood as follows:

$$\text{log}\;\text{odds}=\text{log}\frac{p}{1-p}=\beta_{0}+\beta_{1}x_{1}+\beta_{2}x_{2}+\cdots +\beta_{k}x_{k}$$

*p* = probability of one phenotype; $\text{odds}=\frac{p}{1-p}$
*x_i_* = *i*^th^ biomarker value, *i* = 1, 2, …, *k*.

The left side of logit link function above can be signed as *Y*, and then this function can be simply written as *Y* = β_0_ + β*X*, so it can actually be understood as a linear regression. The phenotype variable in logit could be binary value (e.g., AR vs. non-AR) or multinomial.

## Correlation

Besides machine learning, other mathematical methods have also been employed to establish quantitative biomarker system. Here, we introduce one, kSORT (15), based on Pearson correlation coefficients of multiple genes (12 genes). Genes for kSORT were selected by Lasso and elastic-net. Binary classification of AR vs. non-AR was based on accumulated scores. These scores were accumulated from running correlation for 13 times with 12 gene panels. The score was assigned as 1 (if greater correlation to AR) or −1 (if greater correlation to non-AR). After 13 runs, the final score of a sample would be in the range from −13 to 13. This rank is then used as an index of a risk factor for acute rejection.

## Data Integration Strategies

Many omic experiments have been performed with different platforms by different laboratories. These omics data cannot be treated as a single experiment and a meta-analysis strategy should be applied to integrate these data to get a panel of prioritized genes. Several techniques and theories have been proposed (34, 53, 54). Here, we only briefly described four methods employed in transplant biomarker development.

### Normalization and batch effect removal

For combining small set of microarray data with a big data set, normalization can be performed using Lowess (locally weighted scatterplot smoothing) or Loess (55) (later generalization of Lowess), and then batch effect should be removed. Lowess is a non-parametric regression method for fitting a smoothing curve to a dataset by combining regression models and weights of local neighbors. It splits the microarray intensity curve into a series of windows by a given window size and then performs regression locally with nearest weight to smooth the curve. A larger window size produces a smoother curve, and a smaller window size generates more local variation. After normalized, batch effect can be removed by using empirical Bayes methods (56) before combining the data (57).

### Frozen robust multiarray analysis

Robust multiarray analysis (RMA) is a pre-processing algorithm that pre-processes microarray data by background correction, quantile normalization, and summarization in a modular way by fitting the normalized data with models. It is widely used, but RMA cannot be used in clinical data directly because these data are normally in small batches, and clinical samples are normally processed individually and separately and they are comparable. Therefore, a modified RMA, frozen robust multiarray analysis (fRMA), has been proposed. fRMA computes and freezes probe-specific effects and variances of a dataset, and with new data sets coming, these precomputed and frozen info are used in concert with those from the new coming microarrays to normalize and summarize the data. Thus, it provides a way to combine data analyzed individually or in small batches (53).

**Table 1:** **Primary computational algorithms for transplant biomarker discovery**.

Name	Environment	Features and functions	Limitations	Availability	Web resources
**Biomarker selection**					
Traditional stepwise methods	R/SAS	Stepwise regression	High R^2^, low SE	Free/commercial	www.r-project.org, www.sas.com
LASSO and Elastic-net	R/SAS	Shrinkage and biomarker selection	Model could be saturated for Lasso when simple size is small	Free/commercial	www.r-project.org, www.sas.com
Prediction analysis of microarrays (PAM)	Excel/R	Shrinkage and biomarker selection	Might not improve the overall discriminant accuracy	Free	http://statweb.stanford.edu/~tibs/PAM/
ClaNC	R	Classification and biomarker selection	Limited improvement in discriminant accuracy	Free	http://www.stat.tamu.edu/~adabney/clanc/
Principal component analysis	R/SAS	Classification and biomarker selection	Sometimes it is hard to interpret data	Free/commercial	www.r-project.org, www.sas.com
**Prediction models**					
Linear discriminant analysis (LDA)	R/SAS	Classification and prediction	Linear	Free/commercial	www.r-project.org, www.sas.com
logistic regression	R/SAS	Classification and prediction	Requires large sample size	Free/commercial	www.r-project.org, www.sas.com
**Data integration strategies**					
Normalization and batch effect removal	R	Pre-processing	Might not fit clinical data directly	Free	https://www.r-project.org/
Frozen robust multiarray analysis (fRMA)	R	Pre-processing	Requires large data set and platform limitation	Free	https://www.r-project.org/
P-value meta-analysis	Any/R	Gene prioritization	Significance test only	Free	https://www.r-project.org/
Fold-change meta-analysis	Any/R	Gene prioritization	Fold-change-based effect size only	Free	https://www.r-project.org/

When merging multiple datasets, the raw data (e.g., CEL file for Affymetrix array) are usually used in order to pre-processing all data (e.g., outlier deletion and normalization) with the same algorithm and the similar criteria. If multiple probes exist for a transcript, the mean of probes is used to represent the expression level of this transcript. The genome annotation IDs (e.g., refGene IDs) universal for all platforms are usually employed to combine the data.

### *p*-value meta-analysis

*p*-value meta-analysis uses Fisher’s method to combine the squares of the *p*-values as defined below (34):

$$\chi_{2k}^{2}=-2\sum_{i=1}^{k}\text{log}\left(p_{i}\right)$$

where *k* = experiment number, *p_i_* is devised from each experiment. This *p*-value combination would generate a list of genes with meta *p*-value for each gene. Genes with up- and down-regulation is separated into two groups during combining *p*-value but only one with minimum *p*-value is selected. The meta *p*-value can then further be corrected by multiple hypothesis testing to obtain adjusted *p*-value. The final adjusted *p*-value is used to prioritize genes. Lower in adjusted *p*-value ranked in the top.

### Fold-change meta-analysis

Another prioritized gene method is based on fold-change meta-analysis as defined below (34):

$$f_{\text{meta}}=\frac{\left(f_{1}w_{1}+f_{2}w_{2}+f_{3}w_{3}+\ldots \right)}{\left(w_{1}+w_{2}+w_{3}+\ldots \right)},w_{i}=\frac{1}{\text{var}(f_{i})}$$

where,

*f_i_* = fold-changes in sample *i*;
*w_i_* = reverse variance of the *f_i_*.

It should be noted that meta-analyses might produce different rank prioritizations of genes differentially expressed across studies, and the final biological relevance of the selected genes also becomes important in the final gene selection.

## Conclusion and Future Directions

In order to take our understanding of injury mechanisms in organ transplantations to the next level, integration of molecular measurement data from different experiments and different technologies is required. Furthermore, these integrated data need to be analyzed at a global, systems biology level to identify better diagnostic and therapeutic markers. Predictive biomarkers should cover diverse genetic and epigenetic backgrounds. Clinical and pathology-based variables should be considered as confounding variables during biomarker development. Next-gen sequencing will provide much higher resolution than microarrays to get insights into the diversity of injury and patient-specific responses. With new advances in mathematical theories, a new biomarker system may include many variables from different biology aspects, such as genetics, epigenetics, clinical variables, and pathology.

Biomarker discovery suffers from difficulties in selecting “noise” form “true biology” as this discovery relies on human studies and human samples, which are inherently associated with sample and tissue variation, and often result from the use of multiple measurement platforms, with experimental methodology variations, all of which challenge the process of robust biomarkers discovery. Hence, publications suggest that a set of biomarker that works well for one center or one set of patients based on well-conducted statistical methods may not work as well for another center or a different set of patients with variable demographics or other previously unrecognized clinical confounders. It is also important to recognize that most of the biomarkers in transplantation are still in development, as they do not have the support of robust prospective clinical trials. The biomarkers also face the challenge of their correlation being based on histology as the “gold standard,” a standard that we know is not perfect, as it underdiagnoses alloimmune injury and is not really a predictive measure. Thus, most of the biomarkers in research are really just “associated” with histologic findings. It is still unclear if using these biomarkers will ever actually improve graft survival as this requires the conduct of large clinical trials with long-term follow-up, making this research very expensive and often untenable in clinical practice; thus, most omic studies are underpowered with it comes to predicting graft loss. Biomarkers often do not dictate the type of therapy, but accurate prediction of immune risk may allow for their use as companion diagnostics for specific drugs or for safe immunosuppression minimization. Biomarkers may also not always obviate the need for a biopsy, as often they do not differentiate between infections such as polyoma and rejection as both are associated with graft inflammation. Thus, there may still be the need for confirmatory biopsies for the cause or type of organ injury.

The fragmented and incomplete nature of the existing knowledge bases poses a challenge to achieving these goals, and wider adoption of a policy to submit raw data into public repository should be required by the transplant-related journals. It is also imperative that the next generation of clinician scientists is armed with computational skills that will ensure novel questions continued to be posed and answered, enabled by the proper integration of diverse sources of data.

## Conflict of Interest Statement

The authors declare that the research was conducted in the absence of any commercial or financial relationships that could be construed as a potential conflict of interest.
